# A dual-database bibliometric analysis of post-COVID-19 condition and cognitive dysfunction (2021–2025)

**DOI:** 10.3389/fneur.2026.1844317

**Published:** 2026-09-11

**Authors:** Junlin Pu, Wenjie Chen, Hangcheng Jiang, Hong Yang

**Affiliations:** School of Medical and Life Sciences, Chengdu University of Traditional Chinese Medicine, Chengdu, Sichuan, China

**Keywords:** bibliometric analysis, cognitive dysfunction, post-COVID-19 condition, research hotspots, research trends

## Abstract

**Background:**

Coronavirus disease 2019 (COVID-19), caused by infection with severe acute respiratory syndrome coronavirus 2 (SARS-CoV-2), has posed a substantial global health challenge. Cognitive dysfunction is a prevalent and disabling manifestation of post-COVID-19 condition (PCC). This study aims to conduct a comprehensive bibliometric analysis of research on PCC and cognitive dysfunction.

**Methods:**

Publications on PCC and cognitive dysfunction published between 2021 and 2025 were retrieved from the Web of Science Core Collection (WoSCC) and Scopus. After screening according to inclusion criteria, Bibliometrix (R package), VOSviewer, and CiteSpace were employed for bibliometric and visualization analysis.

**Results:**

746 publications from the WoSCC and 1,117 publications from Scopus were included. Over the past 5 years, the number of publications on PCC-related cognitive dysfunction has exhibited a gradual upward trend. The United States showed the highest publication output, while the University of London demonstrated the highest number of publications among institutions in this network. The Journal of Clinical Medicine showed prominent publication and citation-related indicators in this field. Keyword analysis revealed three primary theme domains, including psychological factors, oxidative stress and neuroinflammation. Keyword burst detection revealed that blood–brain barrier, choroid plexus, and endothelial cells represent emerging research frontiers.

**Conclusion:**

This bibliometric analysis systematically maps the global research landscape and evolving trends in PCC-related cognitive dysfunction. Our findings provide a broad overview of research development, collaboration patterns, and emerging topics, which may help generate hypotheses for future mechanistic investigations and clinical research.

## 1 Introduction

According to statistics from the World Health Organisation (WHO), by the end of December 2025, the number of officially recorded deaths from coronavirus disease 2019 (COVID-19) worldwide had exceeded 7 million (1). COVID-19 is caused by severe acute respiratory syndrome coronavirus 2 (SARS-CoV-2), which is a single-stranded positive-sense RNA virus. The spike protein of SARS-CoV-2 exhibits high binding affinity to the angiotensin-converting enzyme 2 (ACE2) receptor on cell membranes (2, 3). ACE2 in the brain plays a key role in recovery from brain injury, the stress response and memory function (4, 5). Song et al. (6) found that mice overexpressing human ACE2 and infected with SARS-CoV-2 showed significant brain infection. Numerous studies have confirmed that SARS-CoV-2 infection can lead to the development of neurological disorders (7–9). Although COVID-19 has been effectively controlled, there is growing evidence indicating that persistent neuropsychiatric symptoms can emerge following a COVID-19 infection (10–12). Atchison et al. (13) found that, while most patients recover from SARS-CoV-2 infection within 2 weeks, a significant number continue to experience post-COVID-19 symptoms for 12 weeks or longer. Among these patients, 5.2% experience symptoms for more than a year. The most common persistent neuropsychiatric symptoms are mild fatigue, difficulty concentrating, memory impairment and mental health issues (12, 14).

The WHO defines “post-COVID-19 sequelae” as symptoms occurring in individuals with a confirmed or suspected history of SARS-CoV-2 infection, lasting more than 2 months, and not explained by other diagnoses (15). Cognitive dysfunction is a common post-COVID-19 sequela, often manifesting as “brain fog”, which includes attention deficits, memory loss, and executive function impairment, significantly impacting the patient’s quality of life and work performance (16, 17). Recent research by Furman et al. (18) also confirms this, suggesting that COVID-19 may contribute to the development of diseases such as Alzheimer’s disease (AD).

Bibliometrics is the quantitative study of scientific literature. It uses mathematics and statistics to examine the creation, distribution, diffusion and use of scientific publications. This method illustrates the dynamics and patterns of scientific and technological development (19, 20). Although several bibliometric studies have investigated PCC-related research using either the WoSCC or Scopus (21–23), reliance on a single database may introduce coverage-related bias because database indexing policies, journal inclusion criteria, citation tracking systems, and subject classification frameworks differ substantially. WoSCC provides a long-established citation indexing framework with structured citation linkage and curated journal coverage, whereas Scopus generally offers broader journal coverage, including greater representation of multidisciplinary and emerging research areas. Because these databases differ in indexing policies, journal coverage, and classification systems, reliance on a single database may result in incomplete representation of the research landscape. Therefore, the use of both databases can provide complementary perspectives and improve the comprehensiveness of bibliometric analyses. In this study, we adopted a dual-database strategy using WoSCC and Scopus to characterize global publication trends, collaboration patterns, and emerging research hotspots in PCC-related cognitive dysfunction research.

## 2 Methods

### 2.1 Data sources and search strategy

This study was designed as a bibliometric analysis rather than a conventional systematic review or evidence synthesis. Therefore, the objective was to characterize research productivity, collaboration patterns, knowledge structures, and emerging trends rather than to evaluate clinical effectiveness or establish causal relationships. The retrieval of all data was undertaken in accordance with the Preferred Reporting Items for Systematic Reviews and Meta-Analyses (PRISMA) protocol (Supplementary Figure 1 and Supplementary Figure 2). A literature search was conducted in the WoSCC and Scopus to identify studies on PCC and cognitive dysfunction.

In the WoSCC, the following search strategy was applied: TS = (“Post-Acute COVID-19 Syndrome” OR “Post-COVID-19 Syndrome” OR “Post-COVID Condition*” OR “Long COVID” OR “COVID-19 sequelae”) AND TS = (“Cognition Disorders” OR “Brain Fog” OR “Cognitive Dysfunction” OR “Cognitive Impairment” OR “Mental Fatigue” OR “Neuroinflammatory Diseases”).

The search strategy was developed to balance retrieval comprehensiveness and relevance. PCC-related terms were selected based on internationally recognized definitions and commonly used terminology in previous literature (22, 23). Cognitive-related terms were selected to capture both clinically recognized cognitive impairment and patient-reported manifestations. Additional terms were included to identify studies exploring potential mechanisms associated with PCC-related cognitive dysfunction, followed by relevance screening to minimize unrelated records. This search yielded 820 records.

The cognitive-related search terms were designed to capture heterogeneous manifestations of PCC-related cognitive impairment. Terms such as “brain fog” and “mental fatigue” were included because they are commonly used to describe subjective cognitive complaints after COVID-19. The term “Neuroinflammatory diseases” was included to identify studies exploring neuroinflammatory pathways potentially associated with PCC-related cognitive dysfunction.

*The inclusion criteria were as follows*: (1) publications in English; (2) publication between January 1, 2021, and December 31, 2025; and (3) articles and reviews related to PCC and cognitive dysfunction that met the search criteria.

*The exclusion criteria were as follows*: (1) non-article or non-review document types, including conference abstracts, editorials, letters, and other non-original publication formats; (2) publications without relevance to PCC-associated cognitive dysfunction after screening of titles, abstracts, and full texts when necessary; (3) studies focusing solely on neurological inflammatory disorders (e.g., multiple sclerosis or neurodegenerative diseases) without a COVID-19-related context; (4) studies addressing general fatigue-related conditions or mental fatigue unrelated to SARS-CoV-2 infection or PCC. The numbers of excluded records related to these criteria are provided in Supplementary Figures S1 and S2.

After study selection, additional quality control procedures were performed during data preprocessing. Records with incomplete or unusable metadata, including missing keywords, incomplete institutional information, or metadata that could not be correctly recognized during data import, were excluded from subsequent bibliometric analyses. A small number of records were excluded because transcoding errors during database file conversion prevented their metadata from being accurately recognized by the R package “bibliometrix”. Detailed numbers and reasons for exclusions are presented in Supplementary Figures S1 and S2.

**Figure 1:**
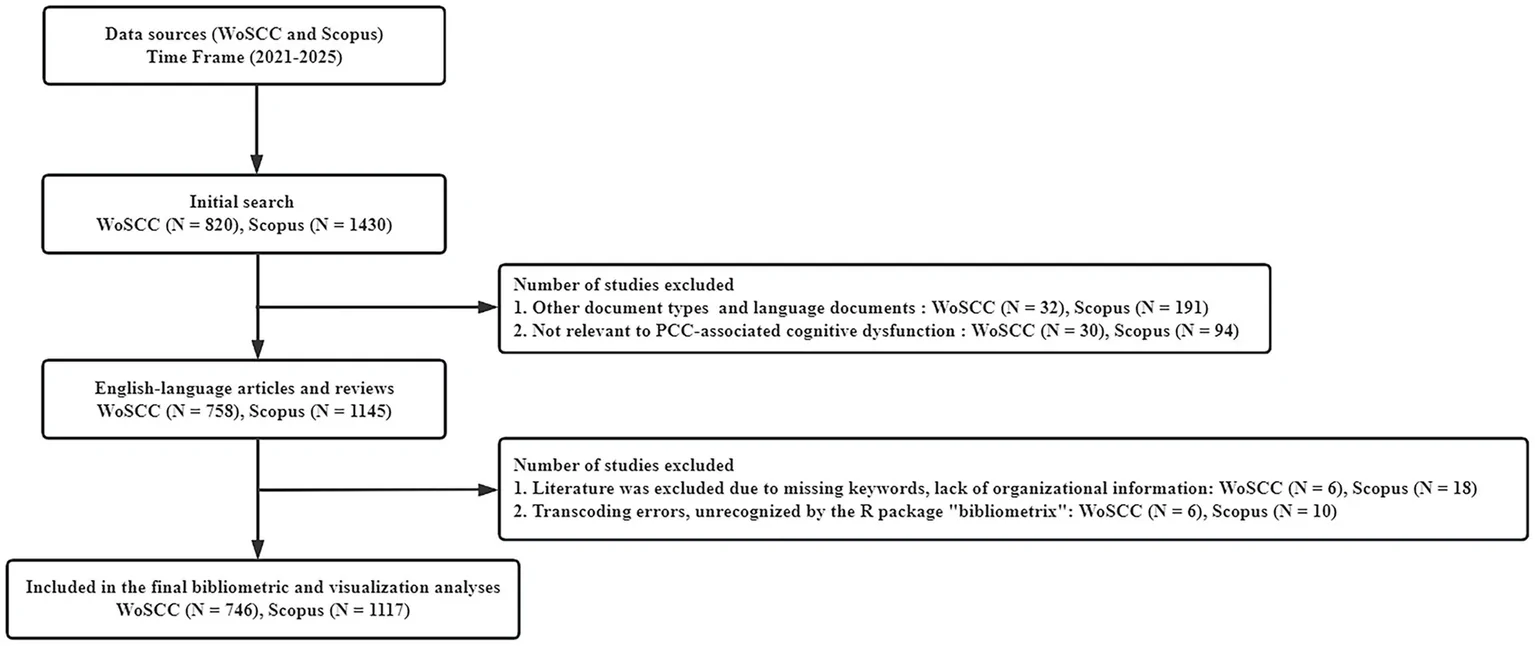
Flowchart of the literature search process.

The literature search was conducted on January 1, 2026. Ultimately, 746 eligible publications were identified from WoSCC. Figure 1 and Supplementary Figure 1 exhibit a flowchart explaining the WoSCC data screening procedure.

In the Scopus, the following search strategy was applied: TITLE-ABS-KEY (“Post-Acute COVID-19 Syndrome” OR “Post-COVID-19 Syndrome” OR “Post-COVID Condition” OR “Long COVID” OR “COVID-19 sequelae”) AND TITLE-ABS-KEY (“Cognition Disorders” OR “Brain Fog” OR “Cognitive Dysfunction” OR “Cognitive Impairment” OR “Mental Fatigue” OR “Neuroinflammatory Diseases”). This search yielded 1,430 records. Using the same inclusion criteria resulted in a final set of 1,117 eligible publications. Figure 1 and Supplementary Figure 2 exhibit a flowchart explaining the Scopus data screening procedure.

The data were collected in text format and included publication and citation counts, titles, journal names, countries, author information, institutions, and keywords for subsequent bibliometric analysis. The final dataset was compiled on January 5, 2026. Supplementary Tables 1 and 2 provide references from WoSCC and Scopus, respectively. Two reviewers independently screened the retrieved records for relevance based on titles and abstracts, with full-text assessment performed when necessary. Disagreements between reviewers were resolved through discussion, and a third reviewer was consulted when consensus could not be reached.

Given that WoSCC and Scopus have different indexing policies, journal coverage, and classification systems, the datasets retrieved from the two databases were analyzed separately rather than merged into a single dataset to preserve database-specific characteristics and minimize potential bias arising from heterogeneous citation and indexing frameworks. To evaluate the complementary contribution of the two databases, records were compared after standardization of publication identifiers, including DOI, title, and publication information. The numbers of overlapping and database-specific records were quantified and are presented in Supplementary Figure 3.

### 2.2 Data analysis and visualization

For the visual analysis of the selected literature, we employed the R package Bibliometrix (version 3.0^1^) (24), VOSviewer (version 1.6.20), and CiteSpace (version 6.4. R1).

As this study aimed to characterize research productivity, collaboration patterns, knowledge structures, and emerging trends, analyses were primarily descriptive and network-based. Statistical hypothesis testing was not performed to compare publication counts across countries, institutions, journals, or authors because bibliometric indicators represent cumulative scientific outputs rather than independent observations suitable for conventional statistical comparisons. The bibliometrix package was used for descriptive bibliometric analysis, including citation metrics, scientific production analysis, and visualization of knowledge structures.

Hirsch index (h-index) was used to highlight the most cited papers in both databases; these papers were identified as the set of “h” papers with “h” or more citations (25, 26). In order to provide a current evaluation of journal prestige and citation influence, the most recent 2025 release of the Journal Citation Reports (JCR) quartiles and Impact Factor (IF) was utilised.

To improve the consistency and comparability of keyword analyses, synonyms, spelling variants, abbreviations, and predefined equivalent terms were normalized before visualization (e.g., “Post-acute COVID-19 syndrome”/“Post-acute sequelae of COVID-19”/“Post-COVID-19 condition”/“Post-COVID condition”/“PASC”/“Long COVID”). Cognitive-related terms, including “Cognitive decline”, “Cognitive deficit”, and “Cognitive dysfunction”, were grouped under a unified cognitive impairment-related category to improve the interpretability of the keyword co-occurrence network.

In addition to keyword normalization, author names and institutional affiliations were harmonized before bibliometric analysis to reduce inconsistencies caused by variations in spelling, abbreviations, and naming conventions. Author names were harmonized based on database records, with attention given to differences in initials and surname formats; potential inconsistencies were manually reviewed when necessary. Institutional names were harmonized by merging clearly corresponding variations of the same institution, including differences in abbreviations, punctuation, and affiliated hospital or university naming formats. These preprocessing procedures were performed before author productivity, institutional collaboration, and co-authorship analyses.

The present study primarily used VOSviewer to conduct the following analyses: institution analysis; journal and co-cited journal analysis; author and co-cited author analysis; and keyword co-occurrence analysis (27).

Keyword analysis was conducted using the ‘Full counting’ method with a minimum occurrence threshold set at 19 occurrences. The threshold selection was guided by Price’s Law and selected to balance the inclusion of relevant keywords with network readability (28). A total of 42 keywords meeting the criteria were included in the visualisation analysis. To minimise the influence of high-frequency keywords on the network structure the association strength normalisation method was applied. Clustering was performed using VOSviewer’s default modularity-based clustering algorithm and all other parameters were maintained at default settings.

Keyword burst detection analysis was conducted using CiteSpace (version 6.4. R1). The parameters were set as follows: the time span was set to January 2021 to December 2025 and the time slicing was set to 1 year; ‘Keyword’ was selected as the node type; the g-index was adopted as the selection criterion with a scaling factor k of 25; Network pruning was performed using the ‘Pathfinder’ and ‘Pruning sliced networks’ methods; all other parameters were set to the software’s default values. Keyword burst detection was performed using the Kleinberg burst detection algorithm implemented in CiteSpace with the number of states set to 2 γ set to 0.1 and minimum duration set to 2

Moreover, Microsoft Excel was employed to calculate annual publication volumes to generate line charts. Given that all data were gathered from public sources and contained no human subjects or private information, ethics committee permission was not necessary.

## 3 Results

### 3.1 Comparison between WoSCC and Scopus datasets

Comparison of the two databases demonstrated substantial but incomplete overlap. Based on standardized DOI, title, and publication information, 640 publications were identified in both databases, whereas 106 and 477 publications were unique to WoSCC and Scopus, respectively (Supplementary Figure 3).

### 3.2 Overview of research on the PCC-related cognitive dysfunction

**Figure 2:**
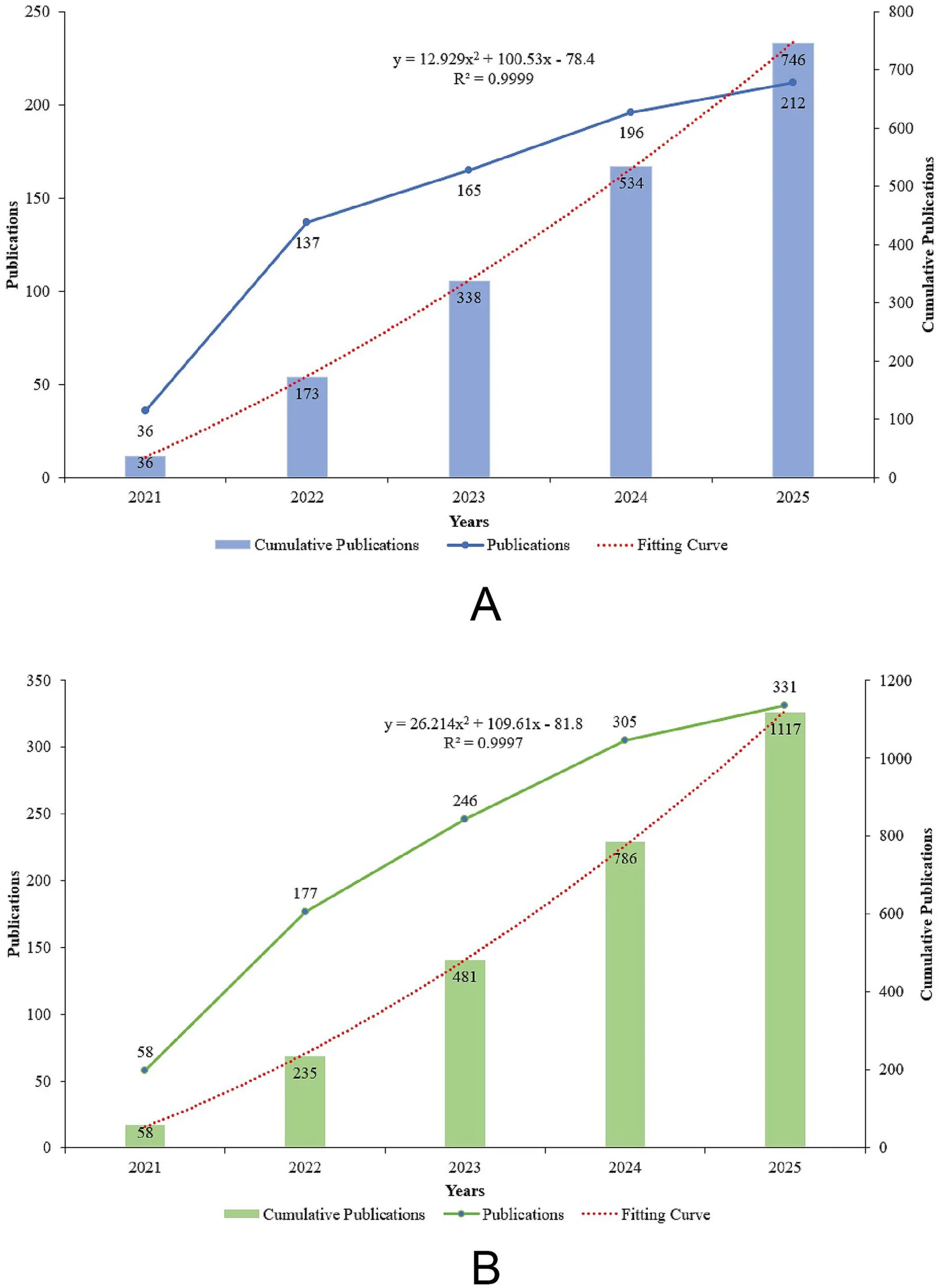
Annual publication trends regarding the role of PCC and cognitive dysfunction in WoSCC **(A)** and Scopus **(B)**.

The fitting curves based on publication numbers (Figure 2A,B) show that the annual number of publications in both databases in the field of PCC and cognitive impairment generally followed an upward trend from 2021 to 2025. Furthermore, the number of papers in both databases was set to peak in 2025. The common trends observed across both databases reflect the academic community’s growing interest in the PCC and cognitive dysfunction.

The most frequently cited article, titled ‘A clinical case definition of post-COVID-19 condition by a Delphi consensus’, was published in 2021 in *Lancet Infectious Diseases* and garnered a total of 2,051 citations. Soriano et al. (29) defined the ‘post-COVID-19 condition’ through a WHO-led Delphi consensus, which provides a foundation for subsequent research into epidemiology, risk factors, clinical characteristics and treatment. The second-most cited paper, titled ‘Characterizing long COVID in an international cohort: 7 months of symptoms and their impact’, was published in 2022 in *Eclinicalmedicine.* It has garnered 1,954 citations. According to the findings of this international online study of 3,762 people with suspected or confirmed COVID-19, long-term COVID is characterized by a variety of sequelae that commonly impair several organ systems, with significant implications for functioning and capacity to work (30). The third most cited article, ‘Long covid-mechanisms, risk factors, and management’, which appeared in *BMJ* in 2021, has also attracted considerable attention, amassing 1,335 citations. The review summarised the impact of long COVID on inpatients and outpatients, and examined the risk factors and potential treatment options for both acute COVID-19 and long COVID (31).

### 3.3 Highly contributive journals

A total of 746 publications were retrieved from WoSCC, which were distributed across 318 academic journals (Supplementary Table 3). The *JOURNAL OF CLINICAL MEDICINE* (IF = 2.9, TP = 19) and *SCIENTIFIC REPORTS* (IF = 3.9, TP = 28), which emerged as the leading journals with an h-index of 10, demonstrate their great influence and significance in the domain (Supplementary Table 5). In Scopus, 1,117 publications were identified, spanning 508 academic journals (Supplementary Table 4). As summarized in Supplementary Table 6, the *JOURNAL OF CLINICAL MEDICINE* (IF = 2.9, TP = 23) remained the leading publishing journal with an h-index of 12. In addition, both of the top 20 journals in the two databases featured the *VIRUSES-BASEL*, demonstrating that research into long COVID and cognitive dysfunction is expanding into the field of virology through an interdisciplinary approach.

**Figure 3:**
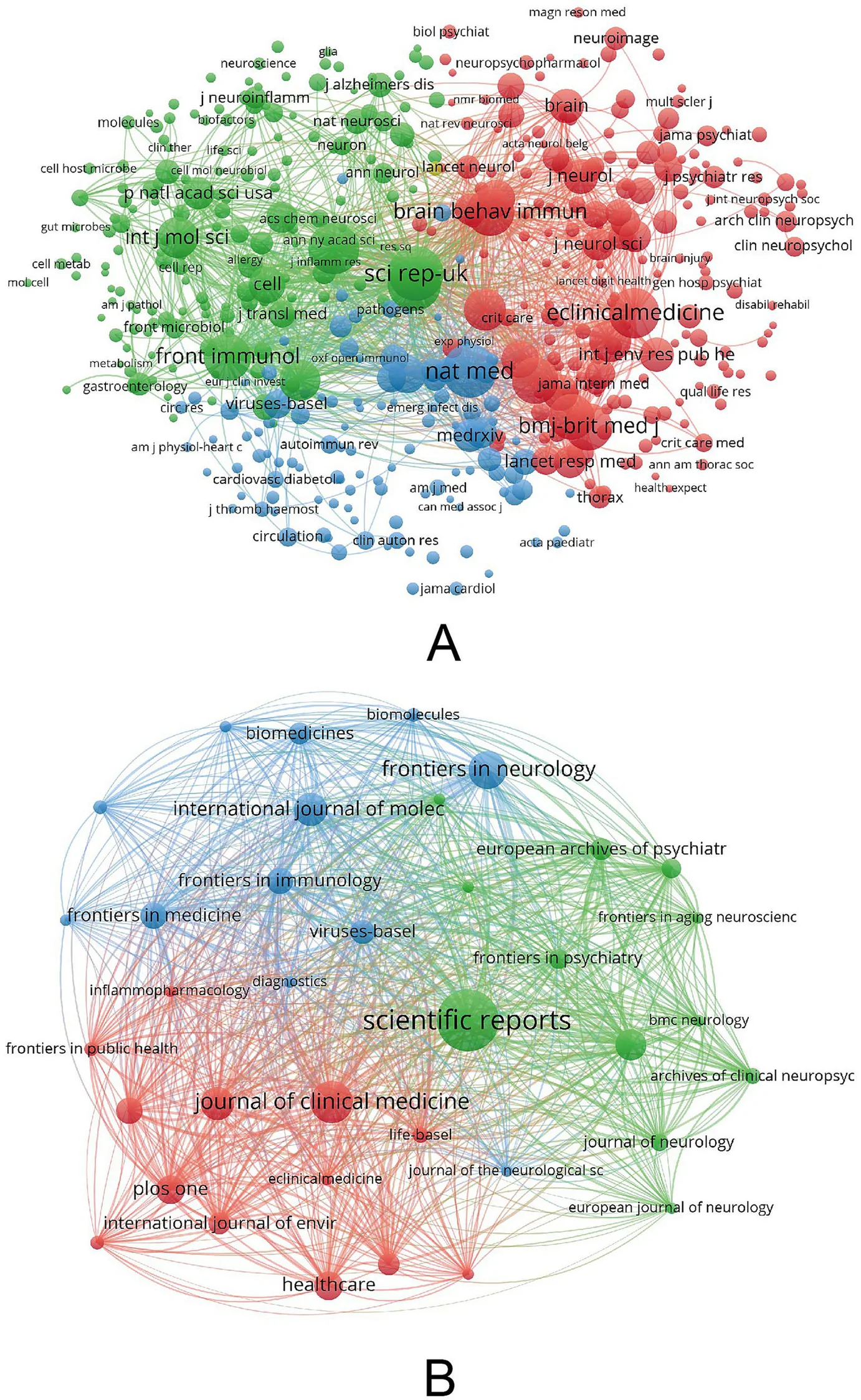
Journal co-occurrence network diagram. **(A)** The frequency with which journals are cited together wit47n the same articles reflects their thematic or topical connections. The colors represent distinct research clusters. **(B)** Journal bibliographic coupling network diagram. The degree of journal linkage is based on shared references cited in their articles, highlighting a common intellectual foundation or research focus. The colors represent different research clusters.

In a keyword co-occurrence network, total link strength (TLS) is defined as the sum of the weights of all links on which a given keyword appears alongside others. A higher value indicates a stronger connection within the network (32). Figure 3A depicts the journals included in the study co-occurrence network diagram associated with PCC and cognitive dysfunction. After analyzing the co-occurrence network, *Frontiers in Immunology* emerged as the highest TLS with 62,418, followed by *Scientific Reports* (TLS = 62,122) and *Nature* (TLS = 59,083). Analyzing the coupling network diagram of journals revealed *Scientific Reports* (TLS = 8,447), *JOURNAL OF CLINICAL MEDICINE* (TLS = 6,828) and *Brain Sciences* (TLS = 6,385) as the top 3 journals by TLS (Figure 3B).

### 3.4 Publication performances: countries

**Figure 4:**
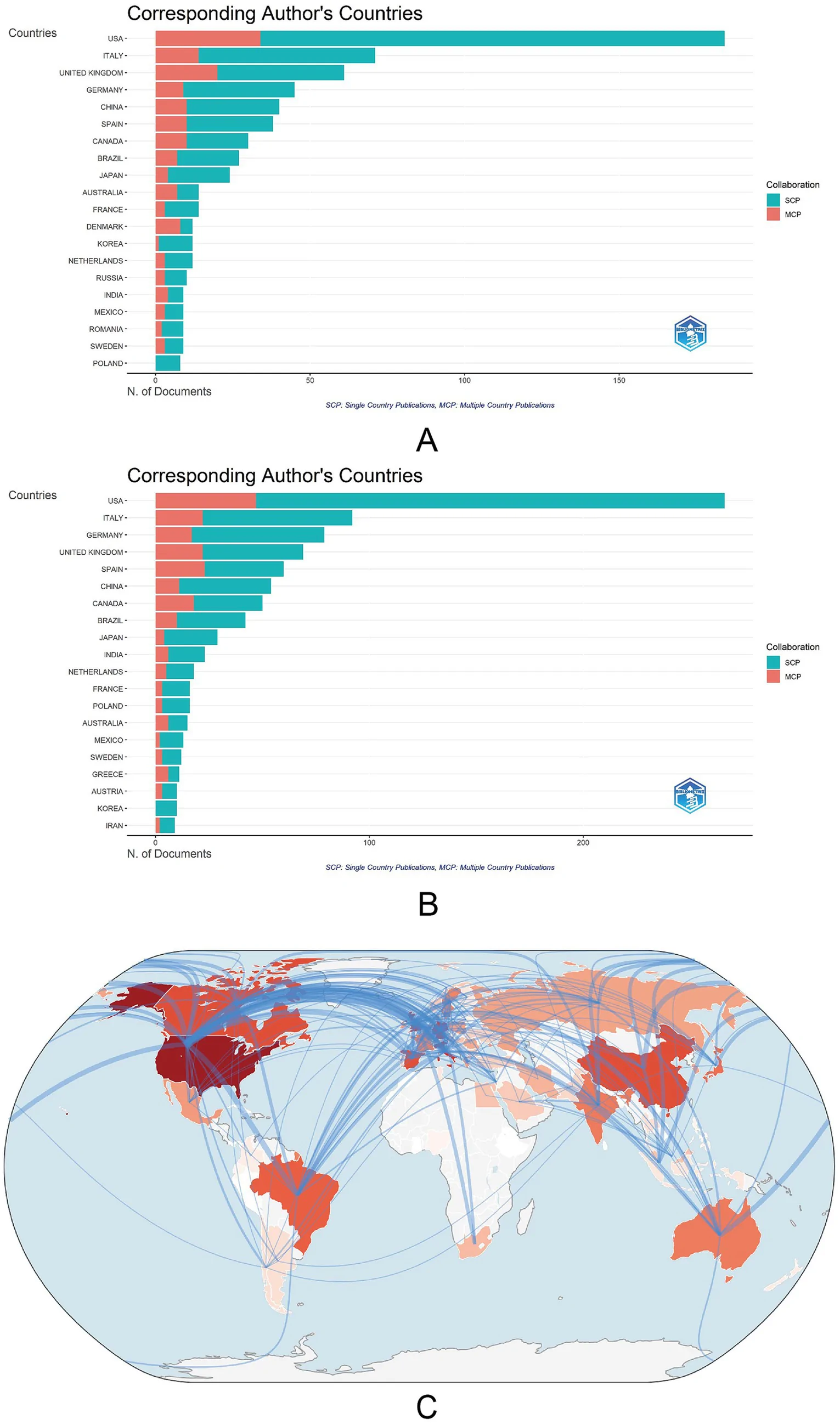
Distribution of corresponding author’s publications by country in WoSCC **(A)** and Scopus **(B)**. MCP, multiple country publications; SCP, single country publications. International collaboration map. **(C)** The color segmentation includes red (indicating publications) and gray (indicating no publications). The thickness of the blue lines represents the number of co-published papers. The color intensity corresponds to the number of publications.

Based on corresponding author affiliations, the United States showed the highest publication output in both databases. In WoSCC, the United States published 184 papers, followed by Italy (*n* = 71) and the United Kingdom (*n* = 61). Similarly, in the Scopus database, the United States (*n* = 266) ranked first in publication output, followed by Italy (*n* = 92) and Germany (*n* = 79) (Figure 4 and Supplementary Table 7–8).

Figure 4C depicts the global collaboration map indicating international cooperation in the field of PCC and cognitive dysfunction. Among the 76 countries that have engaged in at least one paper, the United States established the most collaborations with other countries (244), followed by the United Kingdom (153) and Italy (103).

### 3.5 Publication performances: authors

Supplementary Table 9 shows the publication and citation characteristics of high-impact authors in the field of long COVID and cognitive dysfunction research at WoSCC. Among the high-impact authors, Franke C showed prominent academic influence, with 8 publications and 184 total citations since 2021. His h-index of 7 suggests that his work has gained relatively broad and stable recognition within the field. Followed by Furlanis G, who has produced 7 publications since 2022, accumulating 151 citations. In the Scopus database, DELGADO-ALONSO C, FRANKE C, FURLANIS G, KORALNIK IJ, MANGANOTTI P and MATIAS-GUIU JA were all identified as prominent authors with an h-index of 7, indicating these authors have established a certain degree of academic influence in the field of research into long COVID and cognitive dysfunction (Supplementary Table 10).

**Figure 5:**
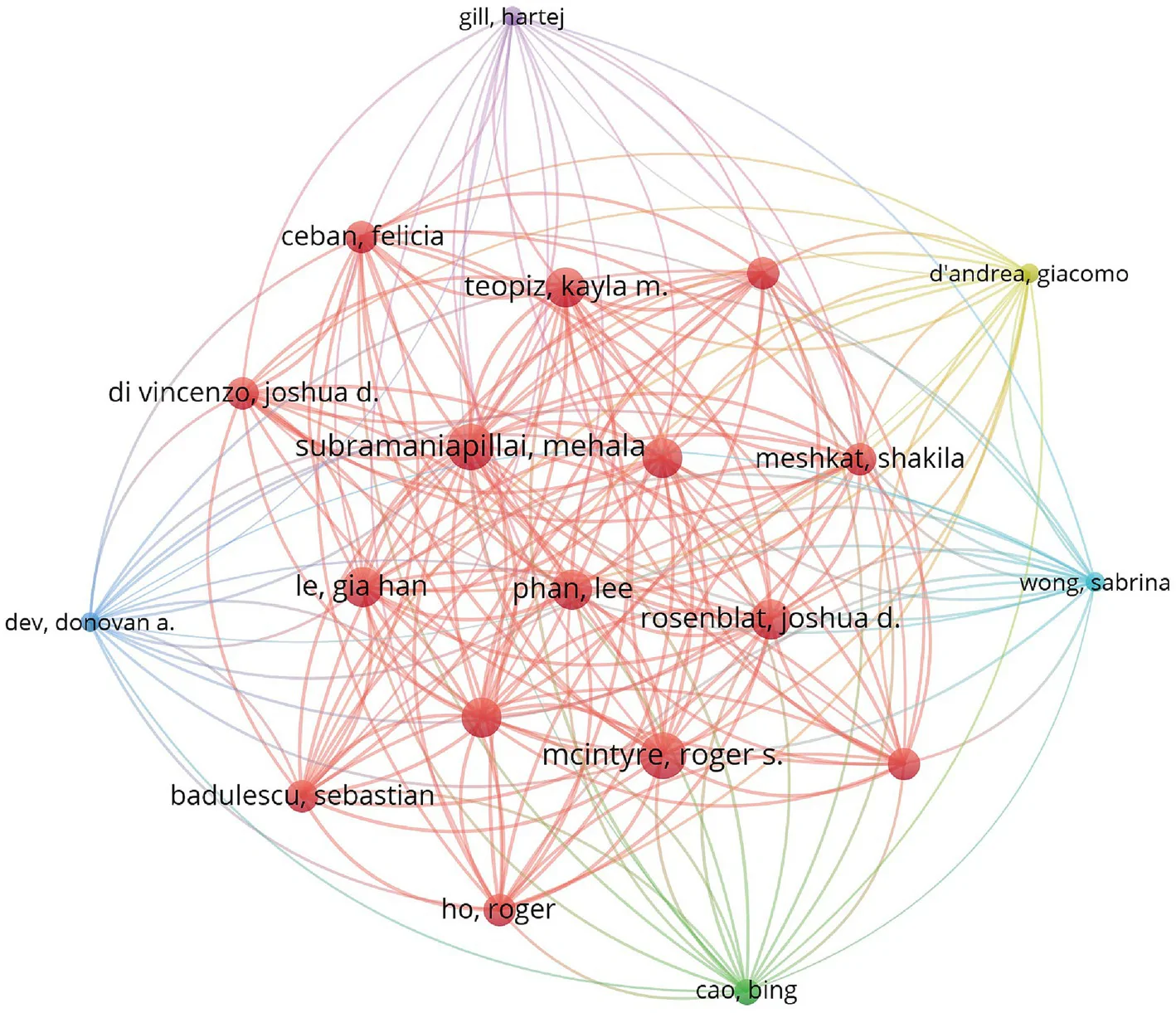
A visualization map depicting collaboration among different authors. Nodes represent authors, with size corresponding to publication count. Links represent co-authorships, with thickness reflecting the strength of collaboration. Colors represent different research clusters. Link strength in collaboration networks quantifies the frequency of co-authorship between authors, indicating the level of collaboration.

Figure 5 shows a visualization map demonstrating collaboration among various authors. McIntyre RS and Subramaniapillai M emerged as the most notable authors, both having the highest TLS (94). Rosenblat JD and Teopiz KM followed closely behind, both with a TLS of 89.

### 3.6 Publication performances: institutions

**Figure 6:**
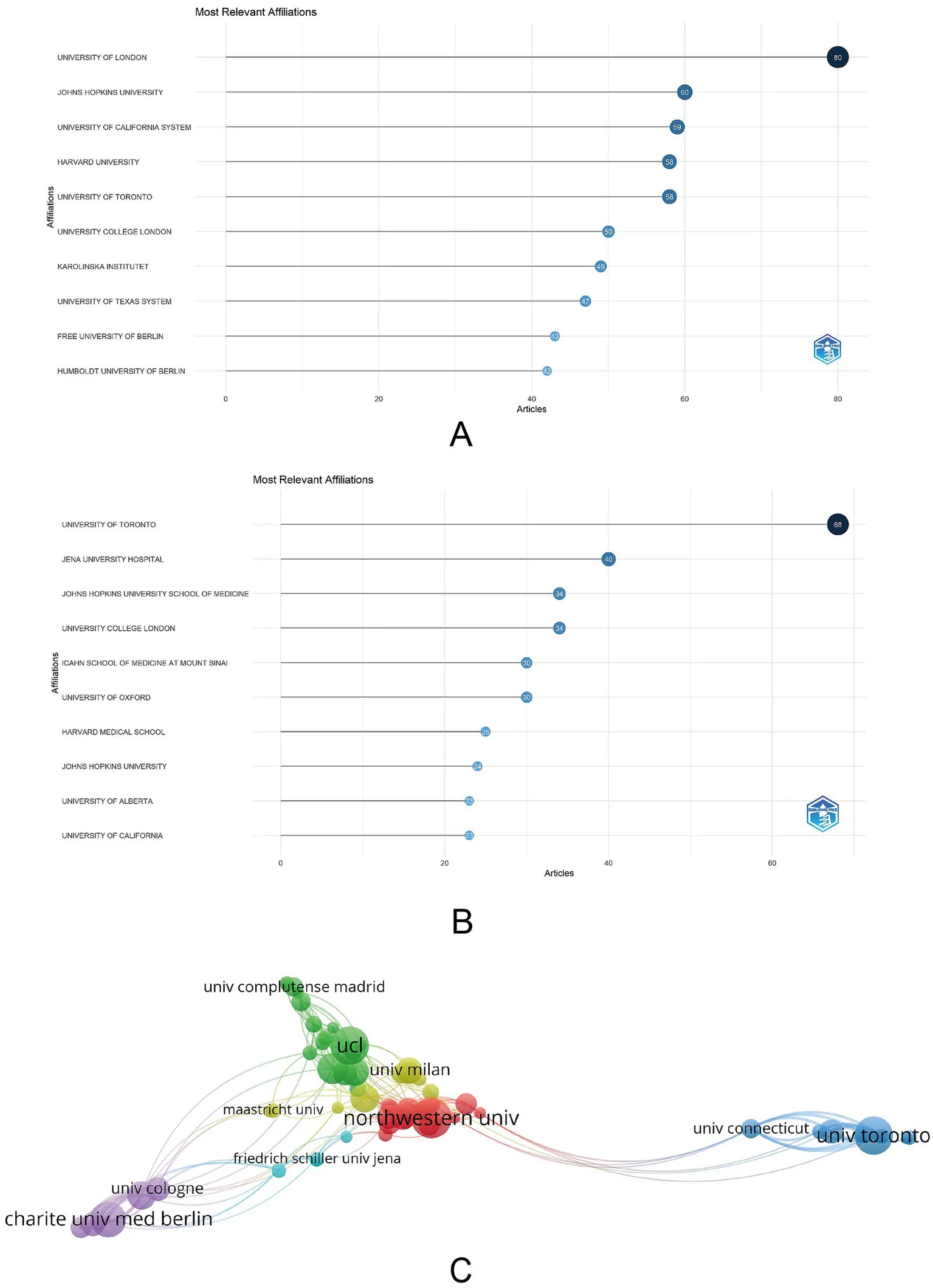
Top 10 institutions by article count and rank in WoSCC **(A)** and Scopus **(B)**. Circle size corresponds to article count. Darker shades indicate higher ranks. **(C)** Visualization map based on WoSCC depicting collaboration among different institutions. Nodes represent institutions, with size corresponding to publication count. Links represent co-authorships, with thickness indicating the strength of collaboration. Colors represent different research clusters.

In WoSCC, the University of London was the most productive institution with 80 publications, closely followed by Johns Hopkins University (*n* = 60) and the University of California System (*n* = 59; Figure 6A). In the Scopus database, the University of Toronto (*n* = 68) ranked first in publication output, followed by Jena University Hospital (*n* = 40) and Johns Hopkins University School of Medicine (*n* = 34; Figure 6B). Institutions with five or more publications were subjected to a co-authorship analysis (Figure 6C). Charité—Universitätsmedizin Berlin, with 15 documents and 572 citations, had the highest TLS of 51, reflecting that it served as a major collaborative hub in the WoSCC database. The German Center for Neurodegenerative Diseases, with 12 documents and 369 citations, had a TLS of 49. The University of Toronto, with 16 documents and 3,363 citations (TLS = 43), underscores its strong influence and close collaborative connections within this research field.

### 3.7 Keyword co-occurrence

**Figure 7:**
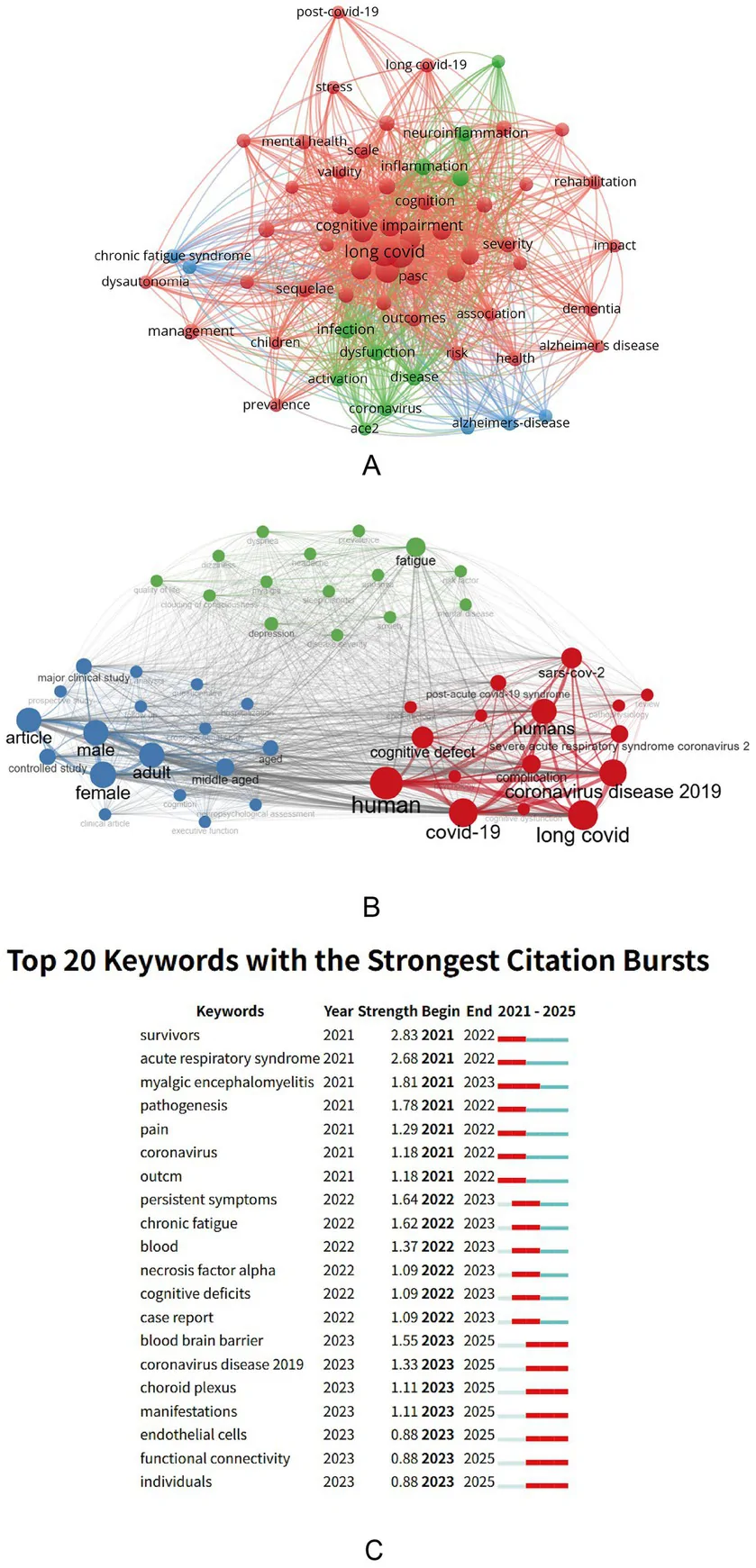
**(A)** Visual analysis of the keyword co-occurrence network. Each node represents a keyword, with its size corresponding to its frequency of occurrence. Links between nodes represent co-occurrence within the same documents, with thicker lines indicating stronger associations. Link strength measures the frequency of co-occurrence between keywords. Colors represent different research clusters. **(B)** Keyword co-occurrence network graph (Scopus; R bibliometrix). Each node represents a keyword, with its size corresponding to its frequency of occurrence. Links between nodes represent co-occurrence within the same documents, with thicker lines indicating stronger associations. Link strength measures the frequency of co-occurrence between keywords. Colors represent different research clusters. **(C)** The top 20 keywords with the strongest citation bursts. The blue lines represent the period, and the red lines indicate the burst periods of the keywords.

Keyword co-occurrence analysis in WoSCC identified three major clusters. The first cluster mainly represented psychological and clinical impacts of PCC including mental health-related concepts. The second cluster focused on ‘oxidative stress’ ‘mitochondrial dysfunction’ and neurodegenerative-related mechanisms. The third cluster represented neurological mechanisms associated with PCC, including ‘neuroinflammation’ and ‘ACE2-related pathways’ (Figure 7A).

The Scopus database’s keyword co-occurrence analysis revealed 3 major clusters. The blue cluster, representing clinical and population-based research, features keywords like ‘human’, ‘adult’, ‘aged’, ‘controlled study’ and ‘major clinical study’. The green cluster representing the mental health issues faced by people with PCC, constitutes terms such as ‘fatigue’, ‘depression’ and ‘anxiety’. The red cluster, representing biomedical and pathophysiological research on PCC, comprises keywords such as ‘coronavirus disease 2019’, ‘SARS-CoV-2’, ‘cognitive defect’ and ‘complication’ (Figure 7B and Supplementary Table 11).

### 3.8 Keyword burst detection

The keyword burst detection from 2021 to 2025 aimed to trace the dynamic changes and emerging patterns in research development across the field (Figure 7C). The keyword ‘survivors’ experienced a substantial citation burst from 2021 to 2022, showing that studies focused on COVID-19 survivors gained momentum rapidly during this period and became a hot topic. Similarly, ‘myalgic encephalomyelitis’ witnessed a significant burst from 2021 to 2023, demonstrating findings linking PCC to myalgic encephalomyelitis/chronic fatigue syndrome (ME/CFS) and symptoms surged and became a prominent emerging focus between 2021 and 2023. In addition, the keywords ‘blood–brain barrier’, ‘choroid plexus’ and ‘endothelial cells’frequently emerged in publications from 2023 to 2025, indicating that research on PCC increasingly focused on neurovascular and barrier dysfunction—especially damage to the blood–brain and endothelial injury—as key mechanisms underlying neurological and cognitive symptoms.

## 4 Discussion

### 4.1 Comparison with previous bibliometric studies and contributions of the present study

Previous bibliometric studies have provided important insights into the research landscape of COVID-19-related cognitive impairment. Grover et al. (23) analyzed Scopus-indexed publications on post-COVID cognitive impairment from 2020 to 2023 and identified major contributors, knowledge networks, and frequently occurring topics, including COVID-19, cognitive impairment, long COVID, fatigue, and depression. Cheng et al. (22) further investigated coronavirus-related cognitive impairment research using WoSCC data and highlighted neuroinflammation and neuroimmune pathways as important research themes. However, these studies either relied on a single database or included broader coronavirus-related cognitive outcomes rather than specifically focusing on cognitive dysfunction associated with PCC.

In contrast, the present study specifically characterized the knowledge structure and research evolution of PCC-related cognitive dysfunction using a dual-database strategy integrating WoSCC and Scopus. This approach provided complementary perspectives on the global research landscape and reduced the dependence on a single indexing system. Furthermore, by incorporating temporal trend analysis, keyword co-occurrence analysis, and keyword burst detection, our study revealed the dynamic evolution of research priorities from early symptom characterization toward increasing attention to mechanism-oriented investigations. Emerging topics, including blood–brain barrier dysfunction, choroid plexus involvement, and endothelial abnormalities, were identified as potential research directions requiring further experimental and clinical validation.

These findings extend previous bibliometric analyses by providing a more focused evaluation of PCC-associated cognitive dysfunction as a distinct post-infectious neurological research field and by highlighting the evolution of research themes and emerging directions beyond descriptive publication mapping.

### 4.2 Global research landscape and collaboration

The bibliometric analysis of PCC and cognitive dysfunction research revealed continuous growth and emerging trends in the field from 2021 to 2025. The increasing publication output in PCC-related cognitive dysfunction research likely reflects growing scientific interest in this emerging field. The increasing recognition of persistent cognitive symptoms after SARS-CoV-2 infection and the need to understand their underlying mechanisms have stimulated multidisciplinary investigations. Although the global burden of COVID-19 provides an important clinical context, bibliometric findings should be interpreted as reflecting changes in research activity rather than direct changes in disease burden.

Beyond the observed increase in publication volume, the evolution of PCC-related cognitive dysfunction research indicates a transition from early characterization of persistent symptoms toward mechanistic investigations. The identified thematic clusters suggest that current research is increasingly integrating clinical manifestations with biological pathways, particularly neuroinflammation, oxidative stress, and neurovascular dysfunction.

The leading contribution of the United States may reflect multiple factors, including substantial research investment, established clinical research infrastructure, and extensive multidisciplinary collaboration networks. However, these bibliometric patterns should be interpreted as differences in research activity rather than direct indicators of scientific superiority.

It should be noted that bibliometric rankings reflect observed publication and citation patterns rather than statistically tested differences or causal relationships. Therefore, variations in publication output among countries, institutions, and journals should be interpreted as descriptive differences in research activity, collaboration patterns, and publication characteristics within the analyzed datasets rather than as evidence of superior research performance.

Furthermore, the significant contribution of the United Kingdom, particularly the University of London, may result from its early governmental research initiatives, strong epidemiological expertise, and coordinated national response to PCC investigations. Authors like Franke C have made major academic contributions, most likely owing to their strong professional background, access to high-level research resources, and engagement in broad academic collaborations. Franke C et al. (33) evaluated the efficacy of methylprednisolone on memory satisfaction and cognitive function in patients with post-COVID syndrome (PCS) complicated by cognitive impairment through the post-COVID-19 immune treatment (PoCoVIT) randomised controlled trial protocol, demonstrating the potential key role of immune-mediated mechanisms in long-COVID cognitive impairment. It also provided an important methodological foundation for subsequent large-scale RCTs on immunosuppressive therapy and mechanistic biomarker studies. This study has had a substantial influence on epidemiological trends, which encouraged future research to focus on the relationship between abnormal immune-mediated processes and the severity of cognitive impairment in PCC, thereby driving the continued development of the literature in this field.

The differences in publication counts and rankings between WoSCC and Scopus should be interpreted in relation to database construction rather than as contradictory estimates of research performance. Under the present search strategy, Scopus retrieved more publications than WoSCC, including 477 Scopus-specific records compared with 106 WoSCC-specific records. The additional records indexed by Scopus may increase the representation of journals and institutions captured within its broader multidisciplinary coverage, thereby influencing publication-based rankings. Conversely, WoSCC rankings reflect the selective set of sources indexed within its citation framework.

Institutional rankings may also be affected by differences in affiliation metadata and aggregation practices. Umbrella university systems, individual campuses, medical schools, and affiliated hospitals may be recorded or consolidated differently across databases. Although institutional names were standardized during data preprocessing, these structural differences could not be completely eliminated. Therefore, the University of London ranking first in WoSCC and the University of Toronto ranking first in Scopus should not be interpreted as conflicting findings or definitive institutional hierarchies, but rather as database-sensitive variations influenced by indexing and affiliation practices.

Importantly, the comparison between WoSCC and Scopus provides insights at two complementary levels. Consistent findings across the two databases—including the upward trend in publication output and the dominant contribution of the United States—may be regarded as relatively robust findings supported by both databases. By contrast, discrepancies in publication counts, journal rankings, and research themes should be interpreted in the context of database-specific characteristics.

Differences in journal coverage may affect publication counts and journal rankings because databases may include different sets of journals, particularly in multidisciplinary and emerging research areas. Differences in indexing practices may influence retrieval results because the inclusion and organization of publications within each database depend on their respective indexing frameworks, which may lead to variations in the number and characteristics of identified records. In addition, differences in subject classification systems may affect the interpretation of research themes and knowledge structures, as the same publication may be assigned to different subject categories across databases. Therefore, discrepancies observed between WoSCC and Scopus should be considered database-specific variations resulting from differences in coverage, indexing, and classification systems rather than direct contradictions in the research landscape.

### 4.3 Research hotspots from keyword analysis

The identified keyword clusters demonstrate the multidimensional nature of PCC-related cognitive dysfunction research. Early studies primarily focused on symptom burden and psychological consequences, whereas subsequent research increasingly explored biological mechanisms, including oxidative stress, mitochondrial dysfunction, and neuroinflammatory pathways. This transition suggests a gradual shift from descriptive clinical observation toward mechanism-oriented investigation.

Keyword co-occurrence analysis of PCC and cognitive dysfunction identified three primary thematic clusters. One of these clusters concentrates on the physical and psychological impacts of PCC. The other examines oxidative stress and mitochondrial dysfunction in the context of long COVID and cognitive dysfunction and the last emphasises neurological effects. High-frequency terms such as ‘mental health’ reflect the impact of psychological factors on the quality of life of long COVID patients whereas keywords such as ‘chronic fatigue syndrome’ and ‘oxidative stress’ highlight the mechanistic basis of the neurological symptoms of long COVID. ‘Infection’ ‘neuroinflammation’ and ‘ACE2’ suggest that viral infection and the resulting neuroinflammation may be the core drivers of the neurological symptoms of long COVID. Fekete et al. (34, 35) have confirmed that COVID-19 patients experience a hyperinflammatory syndrome with significant increases in cytokine levels such as TNF-α and IL-6. As IL-6 levels rise neurons no longer express the IL-6R receptor subtype but instead express the soluble interleukin-6 receptor (sIL-6R) subtype which binds to IL-6 and activates the membrane-bound gp130 (36, 37). This process known as trans-signalling has been shown in previous studies to be associated with cognitive dysfunction in mice induced by lipopolysaccharide (LPS)-induced inflammation (38).

Keywords like ‘human’ ‘adult’ ‘aged’ and ‘major clinical study’ reveal an increased emphasis on clinical and epidemiological validation. Evidence from a population-based analysis conducted by Araújo et al. (39) confirmed that the prevalence of cognitive impairment among individuals who had been infected with SARS-CoV-2 was significantly higher 2 years later than among those who had not been infected; cognitive impairment was more common and more severe among those who had been hospitalised and had a more severe illness (OR 5.41 95% CI: 1.54 19.03). This suggests that long COVID may accelerate the natural process of cognitive ageing. Although interventional studies remain limited emerging evidence suggests that gut probiotics may alleviate some cognitive symptoms (40, 41). Ma et al. (42) conducted a meta-analysis showing that probiotics can significantly improve cognitive function with the effects being particularly pronounced when using a single strain and for interventions lasting ≤12 weeks.

### 4.4 Emerging topics

The emergence of ‘blood–brain barrier’, ‘choroid plexus’ and ‘endothelial cells’ related topics between 2023 and 2025 indicates increasing attention toward neurovascular mechanisms in PCC-related cognitive dysfunction. These topics represent promising hypotheses rather than established mechanisms and require further experimental and clinical validation.

The blood–brain barrier (BBB) serves a protective role in preventing viral damage to brain tissue and safeguarding neural function (43). SARS-CoV-2 antigens have been detected in the endothelial cells of both human and mouse blood vessels. Wenzel et al. (44, 45) have confirmed that SARS-CoV-2 can traverse the endothelial cell layer of BBB models by vitro experiments. Studies have also shown that SARS-CoV-2 can infect choroid plexus epithelial cells and enter the central nervous system (CNS) via the blood-cerebrospinal fluid barrier (B-CSFB) (46). Once the virus invades brain tissue, it continues to infect cells through its neurotropic effect, which may lead to cellular senescence (47, 48). The interaction of SARS-CoV-2 with dopaminergic neurons, resulting in their senescence, may be one of the causes of cognitive dysfunction (49). Consequently, these emerging topics highlight potential biological pathways that warrant further investigation. Future experimental and clinical studies are needed to determine the biological significance and potential clinical relevance of blood–brain barrier integrity, choroid plexus dysfunction, and endothelial inflammatory responses in PCC-related cognitive impairment.

## 5 Conclusion

This study provides a comprehensive bibliometric overview of the global research landscape of PCC-related cognitive dysfunction from 2021 to 2025. Using a dual-database strategy, we characterized publication trends, collaboration patterns, and emerging research themes, revealing increasing research attention toward psychological factors, oxidative stress, neuroinflammation, and neurovascular-related topics. These findings reflect current research priorities and may help generate hypotheses for future investigations. However, as a bibliometric analysis, the identified trends do not establish causal mechanisms or clinical effectiveness, and further experimental and clinical studies are required to validate their biological and clinical significance.

## 6 Strengths and limitations

This study provides a comprehensive bibliometric analysis of research trends, key contributors, hot topics, and emerging directions in the field of PCC-related cognitive dysfunction using data from WoSCC and Scopus. Although this approach offers valuable insights into the evolution of knowledge structures and research priorities, several methodological limitations should be considered when interpreting the findings.

First, the descriptive nature of bibliometric analyses should be acknowledged. Differences in publication volume, citation metrics, and collaboration strength among countries or institutions reflect observed research patterns but do not necessarily indicate statistically significant differences, predictive relationships, or causal mechanisms. This study did not incorporate socioeconomic indicators, research investment data, or other country-level determinants that may influence scientific productivity and citation impact. Future studies integrating these factors with advanced modeling approaches may provide further insights into the determinants of research productivity and scientific impact. Furthermore, as a bibliometric study, our findings primarily describe research patterns and knowledge evolution. Although emerging topics may indicate promising directions, they do not directly demonstrate causal mechanisms or clinical effectiveness. Additionally, as a descriptive bibliometric study, our findings characterize patterns of scientific development and knowledge evolution rather than establish causal relationships or biological mechanisms. Although emerging topics identified through keyword analyses, such as neuroinflammation, oxidative stress, blood–brain barrier dysfunction, and endothelial abnormalities, may indicate promising directions for future investigation, they should be considered hypothesis-generating rather than direct evidence of biological mechanisms. Further experimental and clinical studies are required to validate these potential mechanisms and determine their clinical relevance.

Second, although the inclusion of both WoSCC and Scopus improves literature coverage and enables cross-validation of findings, database-specific bias cannot be completely eliminated. The two databases differ in journal selection criteria, indexing policies, subject classification systems, citation tracking mechanisms, and metadata organization, which may influence the retrieval of publications and the ranking of countries, institutions, journals, and authors. Therefore, differences observed between databases should be interpreted as variations in database coverage rather than definitive differences in scientific contribution.

Third, despite our efforts to construct a comprehensive search strategy, the possibility of search bias cannot be entirely excluded, as no single search strategy can capture all relevant literature. However, the combination of broad search terms, dual-database coverage, and manual relevance screening was designed to improve retrieval sensitivity while maintaining relevance.

Some relevant publications may not have been captured because of variations in terminology, indexing practices, or incomplete bibliographic information. Furthermore, this study included only English-language publications, which may have resulted in underrepresentation of high-quality studies published in other languages and may introduce geographic and linguistic bias.

Fourth, citation-based indicators should be interpreted cautiously because citation accumulation is time-dependent. Publications from recent years, particularly those published in 2024–2025, may have insufficient time to accumulate citations and could therefore be underestimated in citation-based analyses. In addition, citation frequency may be influenced by self-citation practices, collaborative networks, and journal-specific citation behaviors. Importantly, citation counts primarily reflect academic visibility and research attention rather than directly representing methodological quality, scientific validity, or clinical importance. Therefore, citation metrics should not be considered as a direct measure of research quality or clinical impact.

Despite these limitations, the dual-database strategy and the consistency of findings across multiple bibliometric analyses enhance the robustness of our conclusions. The identified research trends, hotspots, and emerging themes provide a valuable macro-level overview of PCC-related cognitive dysfunction research and may facilitate future mechanistic and clinical investigations.

## Data Availability

The original contributions presented in the study are included in the article/Supplementary material, further inquiries can be directed to the corresponding author.

## Footnotes

1
https://bibliometric.com/
